# Assessing the genetic association between vitamin B6 metabolism and genetic generalized epilepsy

**DOI:** 10.1016/j.ymgmr.2019.100518

**Published:** 2019-10-11

**Authors:** Remi Stevelink, Faith Pangilinan, Floor E. Jansen, Kees P.J. Braun, International League Against Epilepsy Consortium on Complex Epilepsies, Anne M. Molloy, Lawrence C. Brody, Bobby P.C. Koeleman

**Affiliations:** a Department of Genetics, Center for Molecular Medicine, University Medical Center Utrecht, Utrecht, the Netherlands; b Department of Child Neurology, Brain Center Rudolf Magnus, University Medical Center Utrecht, Utrecht, the Netherlands; c National Human Genome Research Institute, National Institutes of Health, Bethesda, USA; d School of Medicine, Trinity College Dublin, Dublin, Ireland

**Keywords:** Pyridoxine, GGE, GWAS, Genetics, SNP, Pharmacogenetics

## Abstract

Altered vitamin B6 metabolism due to pathogenic variants in the gene *PNPO* causes early onset epileptic encephalopathy, which can be treated with high doses of vitamin B6. We recently reported that single nucleotide polymorphisms (SNPs) that influence *PNPO* expression in the brain are associated with genetic generalized epilepsy (GGE). However, it is not known whether any of these GGE-associated SNPs influence vitamin B6 metabolite levels. Such an influence would suggest that vitamin B6 could play a role in GGE therapy. Here, we performed genome-wide association studies (GWAS) to assess the influence of GGE associated genetic variants on measures of vitamin B6 metabolism in blood plasma in 2232 healthy individuals. We also asked if SNPs that influence vitamin B6 were associated with GGE in 3122 affected individuals and 20,244 controls. Our GWAS of vitamin B6 metabolites reproduced a previous association and found a novel genome-wide significant locus. The SNPs in these loci were not associated with GGE. We found that 84 GGE-associated SNPs influence expression levels of *PNPO* in the brain as well as in blood. However, these SNPs were not associated with vitamin B6 metabolism in plasma. By leveraging polygenic risk scoring (PRS), we found suggestive evidence of higher catabolism and lower levels of the active and transport forms of vitamin B6 in GGE, although these findings require further replication.

## 1 Introduction

Treatment with vitamin B6 can control seizures in a subset of children with early-onset intractable seizures [1]. Such vitamin B6-responsive epilepsy can be caused by mutations in a number of genes, particularly pyridoxal-5′-phosphate oxidase (*PNPO* [[2], [3], [4], [5]]), which is essential to convert pyridox(am)ine-5′-phosphate into the active form of vitamin B6, pyridoxal-5′-phosphate (PLP). In mammals, PLP is a cofactor for >160 different enzymatic reactions, including the metabolism of the neurotransmitters glutamate and GABA [6].

Interestingly, PLP levels are also reduced in some patients with common forms of epilepsy [7,8], possibly due to the effects of anti-epileptic drugs [9,10]. Moreover, dietary depletion of PLP can induce seizures and epileptiform EEG abnormalities in healthy individuals [11,12]. PLP treatment can reduce seizure frequency in some refractory epilepsy patients without documented pathogenic variants [7,13].

Our recent genome-wide association study (GWAS) of genetic generalized epilepsy (GGE) confirmed and strengthened a genome-wide significant association between GGE and a haplotype containing *PNPO* as the most likely causal gene [14]. We found that GGE-associated SNPs alter expression of *PNPO* in the dorsolateral prefrontal cortex [14], suggesting that altered vitamin B6 metabolism might be involved in the pathophysiology of GGE. If so, metabolic pathways involving vitamin B6 might be a therapeutic target. However, it is unknown whether SNPs that influence metabolite levels in blood also predispose to GGE. Likewise, it is not known if GGE associated SNPs are associated with changes in vitamin B6 levels in blood. We sought to answer both of these related questions.

Here, we assessed the genetic association of vitamin B6 metabolites with GGE, utilizing data from two independent large studies, one which compared genetic variants between people with and without epilepsy [14] and the other which evaluated genetic influences of blood vitamin B6 metabolites in healthy individuals [15]. Our previously reported GWAS on 2232 healthy individuals assessed the influence of genetic variants on three different pyridoxine metabolite concentrations measured in blood: PLP, the cell-membrane transport form pyridoxal (PL), and the catabolite pyridoxic acid (PA) [15]. To fully capture genetic contribution to vitamin B6 metabolism, we repeated these genome-wide analyses with imputed genotypes and examined two additional, derived markers of pyridoxine metabolism [6]: the ratios $\frac{\mathrm{PLP}}{\mathrm{PL}}$ (“PLP:PL”) and $\frac{\mathrm{PA}}{\mathrm{PLP}+\mathrm{PL}}$ (“PAr index”). We then used two approaches to determine whether genetic contribution to vitamin B6 metabolism or GGE might be reciprocally informative. First, we assessed whether the GGE-associated SNPs that alter *PNPO* expression are associated with these 5 measures of pyridoxine metabolism. Second, we utilized polygenic risk scoring (PRS) to assess whether the SNPs that influence pyridoxine metabolism are also associated with GGE by comparing PRS for the five measures of pyroxidine metabolism between 3122 people with GGE and 20,244 controls.

## 2 Methods

### 2.1 Subjects

A sample of 2232 healthy individuals from the Trinity Student Study (TSS) were studied to assess genetic variants that influence pyridoxine metabolism. TSS participants are ethnically Irish people aged 18 to 28 years without any serious medical conditions [15,16].

A subset of 3122 non-related subjects with GGE and 20,244 controls from the epilepsy GWAS of the ILAE Consortium on Complex Epilepsies were studied for PRS analyses [14]. These consisted of a subset of the subjects with European ancestry drawn from the more ethnically diverse subjects in the original GWAS. Moreover, the TSS, which served as a control cohort for the original epilepsy GWAS, was excluded from the current PRS analyses. Approval was obtained by all relevant institutional review boards and all study participants provided written informed consent.

### 2.2 Measurement of pyridoxine metabolism

We collected non-fasting EDTA blood samples for measurement of B6 vitamer concentrations in the TSS cohort. Samples were centrifuged and plasma was frozen within 3 h of phlebotomy. Details of stability of B6 vitamers under the conditions of collection have been determined [17]. B6 vitamers were measured using liquid chromatography–tandem mass spectrometry, as described previously [15]. The methodology included measurements of the primary B6 vitamers (PLP, PL and PA) plus the less abundant vitamers (pyridoxamine, pyridoxamine phosphate, pyridoxine, pyridoxine phosphate). These latter vitamers were below the limit of detection in most samples and were not included in the GWAS analysis. From the primary B6 vitamers, the ratios $\frac{\mathrm{PLP}}{\mathrm{PL}}$ (“PLP:PL”) and $\frac{\mathrm{PA}}{\mathrm{PLP}+\mathrm{PL}}$ (“PAr index”) were calculated. The amount of vitamin B6 intake from supplements and fortified foods was quantified using a standardized questionnaire in which study participants reported their recent intake from a list of commonly used vitamin supplements. Active nutrient information was obtained for each supplement and converted to μg of nutrient per day as previously described [15,16].

### 2.3 Genotyping and quality control

We performed genotyping, imputation and genotype quality control for the TSS and the ILAE cohorts identically for both cohorts, as described earlier [14]. In addition, we excluded related subjects from each cohort. Genetic relatedness was calculated in the TSS with PLINK [18,19] and in the ILEA using KING [20] and one individual from each pair with 3rd degree or stronger relatedness (kinship coefficient > 0.0442) was retained.

### 2.4 Pyridoxine metabolite GWAS

We repeated the previously published GWAS on log-transformed PLP, PL and PA levels [15]. In addition, we now used imputed genotype data (~6 million SNPs instead of ~750 thousand) and quality control procedures that were the same for TSS and the epilepsy GWAS. We also performed a GWAS on PLP:PL and the PAr index. We performed linear-mixed model association analyses with Emmax [21], and included age, gender and log-transformed vitamin B6 supplement intake as covariates. Genome-wide significance was defined as *P* < 5 × 10^−8^.

### 2.5 *PNPO* eQTL analyses

Summary statistics from the previously published GGE GWAS [14] were used to specifically assess SNPs in the previously found genome-wide significant *PNPO* locus. This locus was defined as the lead SNP (SNP with the lowest *P*-value) and all SNPs that were in linkage disequilibrium with the lead SNP (R^2^ > 0.2) and had a P-value<10^−4^. We next used FUMA [22] to assess which of these variants is significantly associated with *PNPO* expression in blood, using expression quantitative trait loci (eQTL) data from the eQTLgen study (based on RNA-sequencing data from 24,886 whole blood and *n* = 4798 peripheral blood mononuclear cell samples [23]). Finally, we assessed the association *P*-value of these SNPs in the 5 different pyridoxine metabolism GWAS.

### 2.6 Polygenic risk score analyses

We used default settings of PRSice to perform PRS analyses to establish whether people with GGE have different pyridoxine metabolism PRS scores compared to controls. In brief, every SNP was assigned a weight according to its association in the 5 different pyridoxine metabolism GWAS. Individual PRS were than calculated as the sum of weighted effect alleles, standardized using a *Z*-transformation: $\frac{\mathrm{PRS}-\mathrm{mean}\left(\mathrm{PRS}\right)}{\mathrm{SD}\left(\mathrm{PRS}\right)}$. Only high-quality SNPs with a genotype call-rate > 0.99 and a minor allele frequency > 0.01 were used. SNPs were pruned to a subset of uncorrelated SNPs (R^2^ < 0.1) and PRS values were calculated with a range of different *P*-value thresholds from 0.0001 to 0.5, in steps of 0.0005 (default for PRSice). Logistic regression analyses were used to assess whether pyridoxine metabolite PRS scores were significantly different in people with GGE compared to controls, while controlling for 10 principal components of ancestry. The ‘best-fit’ P-value threshold was selected, defined as the PRS with the strongest association with GGE. We corrected for multiple testing by using a conservative significance threshold of *P* < .001, as recommended for PRSice [24]. We calculated the explained variance (Nagelkerke's R^2^) by subtracting the full logistic regression model (PRS + covariates) with the null model (covariates only).

## 3 Results

### 3.1 Genetic variants that influence vitamin B6 metabolite levels

**Fig. 1:**
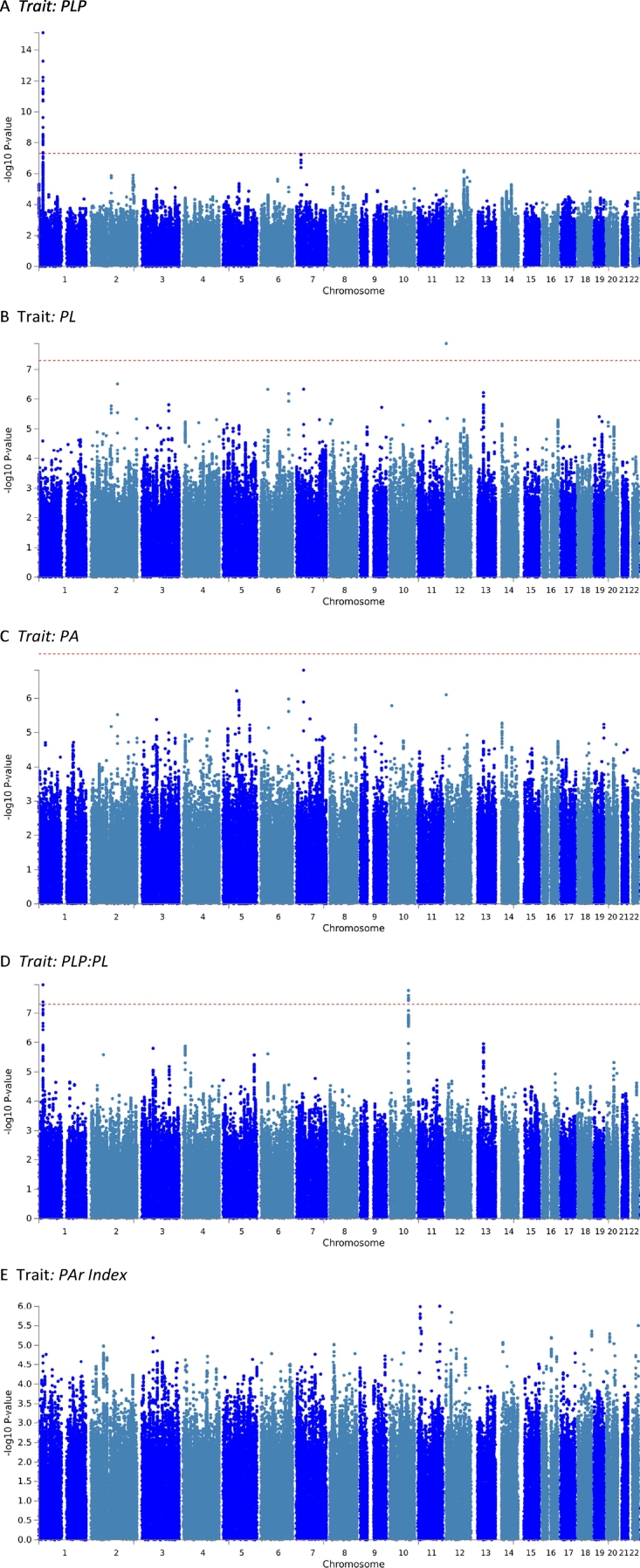
Manhattan plots for each genome-wide association analysis of the five measures of vitamin B6 metabolism. Each genome-wide association analysis was performed using an imputed SNP set and log_10_-transformed values. A) pyridoxal 5′-phosphate (PLP), B) pyridoxal (PL), C) pyridoxic acid (PA), D) PLP:PL ratio, E) PAr index. X-axis: Tested SNPs according to chromosomal position. Y-axis: Negative log_10_-transformed *p*-values. Red line: Genome-wide significance (*p* < 5 × 10^−8^). (For interpretation of the references to colour in this figure legend, the reader is referred to the web version of this article.)

**Fig. 2:**
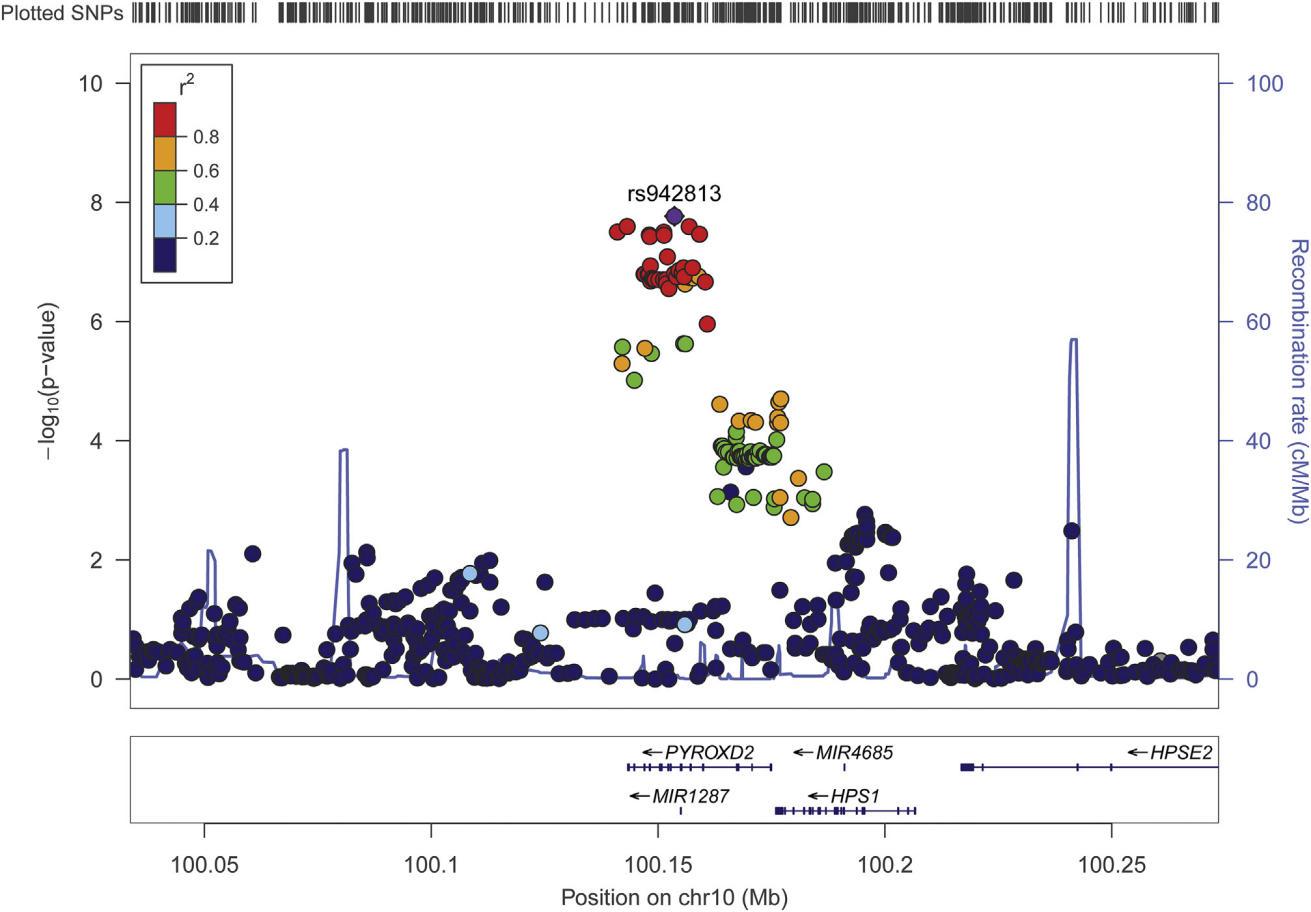
Locusplot of the genome-wide significant locus associated with the PLP:PL ratio. This locus includes a missense variant of the gene PYROXD2 (rs2147896). Chromosomal position including gene annotations are displayed on the X-axis and negative log_10_-transformed *P*-values are displayed on the Y-axis. SNPs are plotted as circles whose colors represent the correlation (linkage disequilibrium) with the lead SNP rs942813.

Our GWAS analyses on vitamin B6 measures (Fig. 1) replicated the genome-wide significant signal at 1p36.12, implicating the ALPL gene (Fig. 1A). This signal was significantly associated with PLP concentrations as well as the PLP:PL ratio (*p* = 7.4*10^−16^ and *p* = 1.1*10^−8^, respectively). In addition, we found a novel PLP:PL locus at 10q24.2 (Fig. 1D), which includes a missense variant of the gene pyridine nucleotide disulfide oxidoreductase domain 2 (PYROXD2, rs2147896; *p* = 3.7*10^−8^; Fig. 2). We note an additional locus associated with PLP (Fig. 1A) in an intergenic region on chromosome 7 that is just under the threshold for genome-wide significance (lead SNP rs61295180, *p* = 5.6*10–8). Last, there is a single imputed SNP on chromosome 12 associated with PL (rs4765900, Fig. 1B), but there is no LD signature and this singleton is likely to be a spurious signal.

### 3.2 Effect of *PNPO* SNPs on vitamin B6 metabolite levels in blood

A genome-wide significant locus identified in the GGE GWAS implicated 84 SNPs around the gene *PNPO* [14]*.* To assess whether these SNPs influence vitamin B6 levels in blood, we leveraged an eQTL database including data from 29,684 subjects and found that all 84 SNPs are associated with *PNPO* expression in blood (eQTL *p*-values between 1.6*10^−85^ and 8.5*10^−8^; see Supplementary Table 1).

We tested these 84 SNPs for association with vitamin B6 metabolites levels and found that only 20 of these SNPs reached nominal significance (*p* < .05) for association with any of the 5 measures of vitamin B6 metabolism (Supplementary Table 2). None survived correction for multiple comparisons, suggesting that GGE-associated variants that influence *PNPO* expression are not associated with concentrations of vitamin B6 metabolites in blood.

### 3.3 Polygenic association of vitamin B6 metabolite SNPs with GGE

To assess whether vitamin B6 metabolism is different in GGE, we first assessed whether the genome-wide significant SNPs from the pyridoxine metabolite GWAS showed an association with GGE. Two out of 44 SNPs from the ALPL locus and none from the PYROXD2 locus from the PLP and PLP:PL GWAS showed a nominally significant association with GGE, but these did not survive correction for multiple testing (Supplementary Table 3).

At just over 2000 individuals, the vitamin B6 metabolite GWAS had limited power to detect associations at the stringent genome-wide significance threshold. It is likely that there are additional, undetected genetic variants with smaller effect sizes than we had the power to detect that influence vitamin B6 metabolite levels. Therefore, we next used PRS analyses to leverage the full distribution of SNPs from the pyridoxine metabolite GWAS, to assess whether people with GGE have a genetic predisposition for different metabolite levels compared to controls. Briefly, polygenic risk scores were generated by determining which genomic SNPs collectively contribute to vitamin B6 metabolite measures in the TSS above a set threshold. These SNPs were used to generate PRS scores in GGE participants and controls to ask whether genetic contribution to vitamin B6 metabolism differs in these groups.

**Fig. 3:**
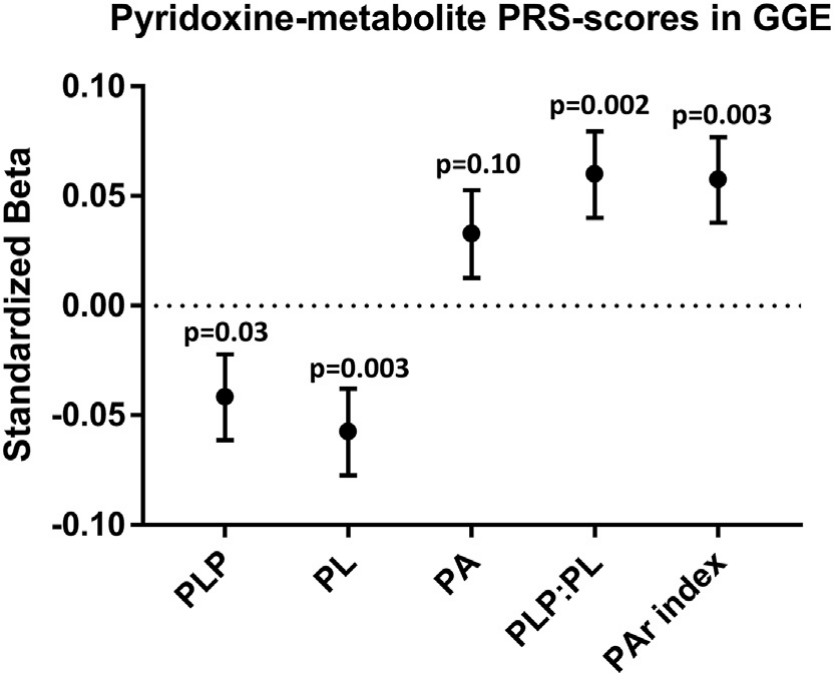
Logistic regression to assess the difference in pyridoxine-related metabolite PRS scores between people with GGE compared to controls. Standardized beta regression coefficients ±standard error are displayed. See Supplementary Table 2 for values. None of the associations reached the significance threshold of *P* < .001 that is recommended for analyses with PRSice.

These polygenic score analyses showed a trend towards lower scores for PLP and PL, but higher scores for PA, PLP:PL and the PAr index in GGE participants (Fig. 3; see Supplementary Table 4 for values). However, these associations did not meet the stringent *P* < .001 threshold that is recommended for analyses with PRSice [24].

## 4 Discussion

In this study, we assessed the genetic association between vitamin B6 metabolism and GGE. We previously found that all 84 GGE-associated SNPs in the *PNPO* locus significantly influenced gene expression of *PNPO*, which is essential to convert vitamin B6 into its active form PLP. In this study, these SNPs were not associated with alterations in vitamin B6 metabolite levels or ratios in blood plasma. However, we cannot rule out the possibility that these SNPs influence vitamin B6 metabolism in the brain or other tissues, where it is needed to convert pyridox(am)ine 5-phosphate to the active form PLP. Indeed, the correlation of PLP as measured in CSF or plasma in children with an intellectual disability was found to be significant but not complete [9], indicating that genetic influence on PLP and the ability to detect it may differ in CSF and plasma. It is possible that GGE-associated *PNPO* SNPs specifically influence metabolism of vitamin B6 in the brain, which could affect neurotransmitter metabolism and influence seizure susceptibility, without altering detectable levels in plasma. However, it is not feasible to collect CSF samples at the scale required for GWAS analyses to ask this more directly. Another caveat to consider is the potentially reductive effect anti-epileptic drugs may have on PLP in the GGE population. This potential influence may have reduced our ability to detect contribution of genetic modifiers of vitamin B6 metabolism to epilepsy in the GGE population.

We reproduced previous GWAS of vitamin B6 levels [15], which confirmed the locus around the gene ALPL, which codes for the enzyme that converts PLP into PL. In addition, by performing a GWAS on PLP:PL, we found a new locus implicating the gene PYROXD2. The same SNPs in this locus are associated with levels of several other metabolites in blood (dimethylamine [25], unknown X-12092 [26,27], caprolactam [28], asymmetric dimethylarginine [29]) and urine (trimethylamine [25,30]) but a role for PYROXD2 in vitamin B6 metabolism has not been previously established. PYROXD2 was initially identified for binding the X protein of human hepatitis B (HBx) in a protein interaction assay [31]. It has been further characterized as having tumor suppressor activity [32], although its exact function remains unknown. Its protein sequence includes sequence conservation with an NAD(P)-binding Rossman-like domain (HomoloGene [33]), which may contribute to a reduction-oxidation activity. The association of PYROXD2 with a measure of vitamin B6 metabolism in the current study may be a helpful clue in elucidating its biological function.

Although we found that the genome-wide significant vitamin B6 metabolism loci were not significantly associated with GGE, we did find suggestive evidence for an association by leveraging the full distribution of SNPs with PRS analyses. These analyses suggested that people with GGE have a genetic predisposition for higher vitamin B6 catabolism (higher PA and PAr index) and lower levels of PLP and PL in blood. Moreover, PRS of PLP:PL was higher in GGE compared to controls, suggesting relatively lower levels of the transport form PL, which is required for delivery to the brain. However, these analyses were limited by a relatively small sample size for the vitamin B6 GWAS (*n* = 2232) and did not meet the stringent *P* < .001 cutoff that is recommended for PRSice. Further studies with a larger sample size are needed to confirm these findings.

In summary, our study did not find evidence for an influence of GGE-associated *PNPO* SNPs on vitamin B6 metabolism in blood, although these SNPs could still have a brain-specific influence on vitamin B6. We found a novel locus that influences the PLP:PL ratio and we found suggestive evidence for increased vitamin B6 catabolism in people with GGE, which needs further replication. However, it is unlikely that genetic differences in vitamin B6 metabolism described here are sufficiently large to be causal in the pathophysiology of GGE or to have direct therapeutic implications.

## Appendix A Supplementary data

Supplementary material Image 1
