# Supplementary material for: Assessing the genetic association between vitamin B6 metabolism and genetic generalized epilepsy
Source: Mol Genet Metab Rep. 2019 Oct 11;21:100518. doi: 10.1016/j.ymgmr.2019.100518 (PMC6796782; doi:10.1016/j.ymgmr.2019.100518)
Supplement: Supplementary file 1 — Supplementary material [file mmc1.docx]

**The International League Against Epilepsy Consortium on Complex Epilepsies**

*Members listed in alphabetical order:*

Bassel Abou-Khalil^1^, Pauls Auce^2, 3^, Andreja Avbersek^4^, Melanie Bahlo^5-7^, David J Balding^8, 9^, Thomas Bast^10, 11^, Larry Baum^12-14^, Albert J Becker^15^, Felicitas Becker^16, 17^ Bianca Berghuis^18^, Samuel F Berkovic^19^, Katja E Boysen^19^, Jonathan P Bradfield^20, 21^, Lawrence C Brody^22^, Russell J Buono^20, 23, 24^, Ellen Campbell^25^, Gregory D Cascino^26^, Claudia B Catarino^4^, Gianpiero L Cavalleri^27, 28^, Stacey S Cherny^13, 29^, Krishna Chinthapalli^4^, Alison J Coffey^30^, Alastair Compston^31^, Antonietta Coppola^32, 33^, Patrick Cossette^34^, John J Craig^35^, Gerrit-Jan de Haan^36^, Peter De Jonghe^37, 38^, Carolien G F de Kovel^39^, Norman Delanty^27, 28, 40^, Chantal Depondt^41^, Orrin Devinsky^42^, Dennis J Dlugos^43^, Colin P Doherty^28, 44^, Christian E Elger^45^, Johan G Eriksson^46^, Thomas N Ferraro^23, 47^, Martha Feucht^48^, Ben Francis^49^, Andre Franke^50^, Jacqueline A French^51^, Saskia Freytag^5^, Verena Gaus^52^, Eric B Geller^53^, Christian Gieger^54, 55^, Tracy Glauser^56^, Simon Glynn^57^, David B Goldstein^58, 59^, Hongsheng Gui^13^, Youling Guo^13^, Kevin F Haas^1^, Hakon Hakonarson^20, 60^, Kerstin Hallmann^45, 61^, Sheryl Haut^62^, Erin L Heinzen^58, 59^, Ingo Helbig^43, 63^, Christian Hengsbach^16^, Helle Hjalgrim^64, 65^, Michele Iacomino^33^, Andrés Ingason^66^, Jennifer Jamnadas-Khoda^4, 67^, Michael R Johnson^68^, Reetta Kälviäinen^69, 70^, Anne-Mari Kantanen^69^, Dalia Kasperavičiūte^4^, Dorothee Kasteleijn-Nolst Trenite^39^, Heidi E Kirsch^71^, Robert C Knowlton^72^, Bobby P C Koeleman^39^, Roland Krause^73^, Martin Krenn^74^, Wolfram S Kunz^45^, Ruben Kuzniecky^75^, Patrick Kwan^12, 76, 77^, Dennis Lal^78^, Yu-Lung Lau^79^, Anna-Elina Lehesjoki^80^, Holger Lerche^16^, Costin Leu^4, 78, 81^, Wolfgang Lieb^82^, Dick Lindhout^36, 39^, Warren D Lo^83^, Iscia Lopes-Cendes^84, 85^, Daniel H Lowenstein^71^, Alberto Malovini^86^, Anthony G Marson^2^, Thomas Mayer^87^, Mark McCormack^27^, James L Mills^88^, Nasir Mirza^2^, Martina Moerzinger^48^, Rikke S Møller^64, 65, 89^, Anne M Molloy^90^, Hiltrud Muhle^63^, Mark Newton^91^, Ping-Wing Ng^92^, Markus M Nöthen^93^, Peter Nürnberg^94^, Terence J O’Brien^76, 77^, Karen L Oliver^19^, Aarno Palotie^95, 96^, Faith Pangilinan^22^, Sarah Peter^73^, Slavé Petrovski^76, 97^, Annapurna Poduri^98^, Michael Privitera^99^, Rodney Radtke^100^, Sarah Rau^16^, Philipp S Reif^101, 102^, Eva M Reinthaler^74^, Felix Rosenow^101, 102^, Josemir W Sander^4, 36, 103^, Thomas Sander^52, 94^, Theresa Scattergood^104^, Steven C Schachter^105^, Christoph J Schankin^106^, Ingrid E Scheffer^19, 107^, Bettina Schmitz^52^, Susanne Schoch^15^, Pak C Sham^13^, Jerry J Shih^108^, Graeme J Sills^2^, Sanjay M Sisodiya^4, 103^, Lisa Slattery^109^, Alexander Smith^78^, David F Smith^3^, Michael C Smith^110^, Philip E Smith^111^, Anja C M Sonsma^39^, Doug Speed^8, 112^, Michael R Sperling^113^, Bernhard J Steinhoff^10^, Ulrich Stephani^63^, Remi Stevelink^39^, Konstantin Strauch^114, 115^, Pasquale Striano^116^, Hans Stroink^117^, Rainer Surges^45^, K Meng Tan^76^, Liu Lin Thio^118^, G Neil Thomas^119^, Marian Todaro^76^, Rossana Tozzi^120^, Maria S Vari^116^, Eileen P G Vining^121^, Frank Visscher^122^, Sarah von Spiczak^63^, Nicole M Walley^58, 123^, Yvonne G Weber^16^, Zhi Wei^124^, Judith Weisenberg^118^, Christopher D Whelan^27^, Peter Widdess-Walsh^53^, Markus Wolff^125^, Stefan Wolking^16^, Wanling Yang^79^, Federico Zara^33^, Fritz Zimprich^74^

1. Vanderbilt University Medical Center, Nashville, TN 37232, USA.

2. Department of Molecular and Clinical Pharmacology, University of Liverpool, Liverpool L69 3GL, UK.

3. The Walton Centre NHS Foundation Trust, Liverpool L9 7LJ, UK.

4. Department of Clinical and Experimental Epilepsy, UCL Institute of Neurology, Queen Square, London WC1N 3BG, UK.

5. Population Health and Immunity Divison, The Walter and Eliza Hall Institute of Medical Research, Parkville 3052, Australia.

6. Department of Biology, University of Melbourne, Parkville 3010, Australia.

7. School of Mathematics and Statistics, University of Melbourne, Parkville 3010, Australia.

8. UCL Genetics Institute, University College London, London WC1E 6BT, UK.

9. Melbourne Integrative Genomics, University of Melbourne, Parkville 3052, Australia.

10. Epilepsy Center Kork, Kehl-Kork 77694, Germany.

11. Medical Faculty of the University of Freiburg, Freiburg 79085, Germany.

12. Centre for Genomic Sciences, The University of Hong Kong, Hong Kong.

13. Department of Psychiatry, The University of Hong Kong, Hong Kong.

14. The State Key Laboratory of Brain and Cognitive Sciences, University of Hong Kong, Hong Kong, China.

15. Section for Translational Epilepsy Research, Department of Neuropathology, University of Bonn Medical Center, Bonn 53105, Germany.

16. Department of Neurology and Epileptology, Hertie Institute for Clinical Brain Research, University of Tübingen, Tübingen 72076, Germany.

17. Department of Neurology, University of Ulm, Ulm 89081, Germany.

18. Stichting Epilepsie Instellingen Nederland (SEIN), Zwolle 8025 BV, The Netherlands.

19. Epilepsy Research Centre, University of Melbourne, Austin Health, Heidelberg 3084, Australia.

20. Center for Applied Genomics, The Children's Hospital of Philadelphia, Philadelphia, PA 19104, USA.

21. Quantinuum Research LLC, San Diego, CA 92101, USA.

22. National Human Genome Research Institute, National Institutes of Health, Bethesda, MD 20892, USA.

23. Department of Biomedical Sciences, Cooper Medical School of Rowan University Camden, NJ 08103, USA.

24. Department of Neurology, Thomas Jefferson University Hospital, Philadelphia, PA 19107, USA.

25. Belfast Health and Social Care Trust, Belfast BT9 7AB, UK.

26. Division of Epilepsy, Department of Neurology, Mayo Clinic, Rochester, MN 55902, USA.

27. Department of Molecular and Cellular Therapeutics, The Royal College of Surgeons in Ireland, Dublin 2, Ireland.

28. The FutureNeuro Research Centre, Dublin 2, Ireland.

29. Department of Epidemiology and Preventive Medicine, School of Public Health, Sackler Faculty of Medicine, Tel Aviv University, Tel Aviv 6997801, Israel.

30. The Wellcome Trust Sanger Institute, Hinxton, Cambridge CB10 1SA, UK.

31. Department of Clinical Neurosciences, Cambridge Biomedical Campus, Cambridge CB2 0SL, UK.

32. Department of Neuroscience, Reproductive and Odontostomatological Sciences, University Federico II, Naples 80138, Italy.

33. Laboratory of Neurogenetics and Neurosciences, Institute G. Gaslini, Genova 16148, Italy.

34. Department of Neurosciences, Université de Montréal, Montréal, CA 26758, Canada.

35. Department of Neurology, Royal Victoria Hospital, Belfast Health and Social Care Trust, Grosvenor Road, Belfast BT12 6BA, UK.

36. Stichting Epilepsie Instellingen Nederland (SEIN), Heemstede 2103 SW, The Netherlands.

37. Neurogenetics Group, Center for Molecular Neurology, VIB and Laboratory of Neurogenetics, Institute Born-Bunge, University of Antwerp, Antwerp 2610, Belgium.

38. Department of Neurology, Antwerp University Hospital, Edegem 2650, Belgium.

39. Department of Genetics, University Medical Center Utrecht, Utrecht 3584 CX, The Netherlands.

40. Division of Neurology, Beaumont Hospital, Dublin D09 FT51, Ireland.

41. Department of Neurology, Hôpital Erasme, Université Libre de Bruxelles, Bruxelles 1070, Belgium.

42. Comprehensive Epilepsy Center, New York University School of Medicine, New York, NY 10016, USA.

43. Department of Neurology, The Children's Hospital of Philadelphia, Philadelphia, PA 19104, USA.

44. Neurology Department, St. James’s Hospital, Dublin D03 VX82, Ireland.

45. Department of Epileptology, University of Bonn Medical Centre, Bonn 53127, Germany.

46. Department of General Practice and Primary Health Care, University of Helsinki and Helsinki University Hospital, Helsinki 0014, Finland.

47. Department of Pharmacology and Psychiatry, University of Pennsylvania Perlman School of Medicine, Philadelphia, PA 19104, USA.

48. Department of Pediatrics and Neonatology, Medical University of Vienna, Vienna 1090, Austria.

49. Department of Biostatistics, University of Liverpool, Liverpool L69 3GL, UK.

50. Institute of Clinical Molecular Biology, Christian-Albrechts-University of Kiel, University Hospital Schleswig Holstein, Kiel 24105, Germany.

51. Department of Neurology, NYU School of Medicine, New York City, NY 10003, USA.

52. Department of Neurology, Charité Universitaetsmedizin Berlin, Campus Virchow-Clinic, Berlin 13353, Germany.

53. Institute of Neurology and Neurosurgery at St. Barnabas, Livingston, NJ 07039, USA.

54. Research Unit of Molecular Epidemiology, Helmholtz Zentrum München - German Research Center for Environmental Health, Neuherberg D-85764, Germany.

55. Institute of Epidemiology, Helmholtz Zentrum München - German Research Center for Environmental Health, Neuherberg D-85764, Germany.

56. Comprehensive Epilepsy Center, Division of Neurology, Cincinnati Children's Hospital Medical Center, Cincinnati, OH 45229, USA.

57. Department of Neurology, University of Michigan, Ann Arbor, MI 48109, USA.

58. Center for Human Genome Variation, Duke University School of Medicine, Durham, NC 27710, USA.

59. Institute for Genomic Medicine, Columbia University Medical Center, New York, NY 10032, USA.

60. Division of Human Genetics, Department of Pediatrics, The Perelman School of Medicine, University of Pennsylvania, Philadelphia, PA 19104, USA.

61. Life and Brain Center, University of Bonn Medical Center, Bonn 53127, Germany.

62. Montefiore Medical Center, Bronx, NY 10467, USA.

63. Department of Neuropediatrics, University Medical Center Schleswig-Holstein (UKSH), Kiel 24105, Germany.

64. Danish Epilepsy Centre, Dianalund 4293, Denmark.

65. Institute of Regional Health Services Research, University of Southern Denmark, Odense 5000, Denmark.

66. deCODE genetics, Reykjavik IS-101, Iceland.

67. Department of Psychiatry and Applied Psychology, Institute of Mental Health University of Nottingham, Nottingham NG7 2TU, UK.

68. Faculty of Medicine, Imperial College London, London SW7 2AZ, UK.

69. Kuopio Epilepsy Center, Neurocenter, Kuopio University Hospital, Kuopio 70029, Finland.

70. Institute of Clinical Medicine, University of Eastern Finland, Kuopio 70029, Finland.

71. Department of Neurology, University of California, San Francisco, CA 94143, USA.

72. University of Alabama Birmingham, Department of Neurology, Birmingham, AL 35233, USA.

73. Luxembourg Centre for Systems Biomedicine, University of Luxembourg, Esch-sur-Alzette L-4362, Luxembourg.

74. Department of Neurology, Medical University of Vienna, Vienna 1090, Austria.

75. Department of Neurology, Zucker-Hofstra Northwell School of Medicine, NY 10075, USA.

76. Department of Medicine, University of Melbourne, Royal Melbourne Hospital, Parkville 3050, Australia.

77. Department of Neuroscience, Central Clinical School, Monash University, Melbourne 3004, Australia.

78. Stanley Center for Psychiatric Research, Broad Institute of Harvard and M.I.T., Cambridge, MA 02142, USA.

79. Department of Paediatrics and Adolescent Medicine, The University of Hong Kong, Hong Kong.

80. Folkhälsan Research Center and Medical Faculty, University of Helsinki, Helsinki 00290, Finland.

81. Genomic Medicine Institute, Lerner Research Institute, Cleveland Clinic, Cleveland, OH 44195, USA.

82. Institut für Epidemiologie Christian-Albrechts-Universität zu Kiel, Kiel 24105, Germany.

83. Department of Pediatrics and Neurology, Ohio State University and Nationwide Children's Hospital, Columbus, OH 43205, USA.

84. Department of Medical Genetics, School of Medical Sciences, University of Campinas (UNICAMP), Campinas, SP 13083-887, Brazil.

85. Brazilian Institute of Neuroscience and Neurotechnology (BRAINN), Campinas, SP 13083-970, Brazil.

86. Istituti Clinici Scientifici Maugeri, Pavia 27100, Italy.

87. Epilepsy Center Kleinwachau, Radeberg 01454, Germany.

88. Division of Intramural Population Health Research, Eunice Kennedy Shriver National Institute of Child Health and Human Development, National Institutes of Health, Bethesda, MD 20892, USA*.*

89. Wilhelm Johannsen Centre for Functional Genome Research, Copenhagen DK-2200, Denmark.

90. School of Medicine, Trinity College Dublin, Dublin 2, Ireland.

91. Department of Neurology, Austin Health, Heidelberg 3084, Australia.

92. United Christian Hospital, Hong Kong.

93. Institute of Human Genetics, University of Bonn Medical Center, Bonn 53127, Germany.

94. Cologne Center for Genomics, University of Cologne, Cologne 50931, Germany.

95. Institute for Molecular Medicine Finland (FIMM), University of Helsinki, Helsinki 0014, Finland*.*

96. The Broad Institute of M.I.T. and Harvard, Cambridge, MA 02142, USA.

97. AstraZeneca Centre for Genomics Research, Precision Medicine and Genomics, IMED Biotech Unit, AstraZeneca, Cambridge CB2 0AA, UK.

98. Department of Neurology, Boston Children's Hospital, Harvard Medical School, Boston, MA 02115, USA.

99. Department of Neurology, Neuroscience Institute, University of Cincinnati Medical Center, Cincinnati, OH 45220, USA.

100. Department of Neurology, Duke University School of Medicine, Durham, NC 27710, USA.

101. Epilepsy-Center Hessen, Department of Neurology, University Medical Center Giessen and Marburg, Marburg, Germany and Philipps-University Marburg, Marburg 35043, Germany.

102. Epilepsy Center Frankfurt Rhine-Main, Center of Neurology and Neurosurgery, Goethe University Frankfurt, Frankfurt 60528, Germany.

103. Chalfont Centre for Epilepsy, Chalfont-St-Peter, Buckinghamshire SL9 0RJ, UK.

104. Department of Endocrinology, Hospital of The University of Pennsylvania, Philadelphia, PA 19104, USA.

105. Departments of Neurology, Beth Israel Deaconess Medical Center, Massachusetts General Hospital, and Harvard Medical School, Boston, MA 02215, USA.

106. Department of Neurology, Inselspital, Bern University Hospital, University of Bern, Bern 3010, Switzerland.

107. Department of Neurology, Royal Children's Hospital, Parkville 3052, Australia.

108. Department of Neurosciences, University of California, San Diego, La Jolla, CA 92037, USA.

109. The Royal College of Surgeons in Ireland, Dublin D02 YN77, Ireland. *.*

110. Rush University Medical Center, Chicago, IL 60612, USA.

111. Department of Neurology, Alan Richens Epilepsy Unit, University Hospital of Wales, Cardiff CF14 4XW, UK.

112. Aarhus Institute of Advanced Studies (AIAS), Aarhus University, 8000 Aarhus, Denmark.

113. Department of Neurology and Comprehensive Epilepsy Center, Thomas Jefferson University, Philadelphia, PA 19107, USA.

114. Institute of Genetic Epidemiology, Helmholtz Zentrum München - German Research Center for Environmental Health, Neuherberg D-85764, Germany.

115. Chair of Genetic Epidemiology, IBE, Faculty of Medicine, LMU Munich 80539, Germany.

116. Pediatric Neurology and Muscular Diseases Unit, Department of Neurosciences, Rehabilitation, Ophthalmology, Genetics, Maternal and Child Health, G. Gaslini Institute, University of Genoa, Genova 16148, Italy.

117. CWZ Hospital, 6532 SZ Nijmegen, The Netherlands.

118. Department of Neurology, Washington University School of Medicine, St. Louis, MO 63110, USA*.*

119. Institute for Applied Health Research, University of Birmingham, Birmingham B15 2TT, UK.

120. C. Mondino National Neurological Institute, Pavia 27100, Italy.

121. Departments of Neurology and Pediatrics, The Johns Hopkins University School of Medicine, Baltimore, MD 21287, USA.

122. Department of Neurology, Admiraal De Ruyter Hospital, Goes 4462, The Netherlands.

123. Division of Medical Genetics, Department of Pediatrics, Duke University Medical Center, Durham, NC 27710, USA.

124. Department of Computer Science, New Jersey Institute of Technology, NJ 07102, USA.

125. Department of Pediatric Neurology and Developmental Medicine, University Children's Hospital, Tübingen 72076, Germany.

Supplementary table 1: Association of 84 SNPs in the GGE GWAS PNPO locus, with gene expression of PNPO in blood. Expression QTL (eQTL) data was assessed in the eQTLGen database. The associations of each SNP with expression of PNPO (eQTL p-value) and with GGE (GGE GWAS p-value) are displayed.

| **SNP** | **Chromosome** | **Position** | **eQTL p-value** | **GGE GWAS p-value** |
| --- | --- | --- | --- | --- |
| rs2597166 | 17 | 46072665 | 1.6E-85 | 1.7E-05 |
| rs113516984 | 17 | 45964442 | 3.8E-37 | 3.5E-06 |
| rs72833479 | 17 | 45960449 | 4.5E-37 | 4.5E-06 |
| rs1986693 | 17 | 46026156 | 5.5E-37 | 2.7E-08 |
| rs12951323 | 17 | 46027565 | 3.3E-36 | 2.6E-08 |
| rs4794321 | 17 | 46028844 | 1.7E-35 | 3.0E-08 |
| rs72823509 | 17 | 46044589 | 5.3E-30 | 6.0E-08 |
| rs72823513 | 17 | 46048920 | 5.3E-30 | 4.8E-08 |
| rs8082043 | 17 | 46073080 | 4.7E-29 | 7.7E-08 |
| rs72833468 | 17 | 45949338 | 5.7E-28 | 2.8E-06 |
| rs4793684 | 17 | 45941864 | 1.4E-27 | 1.4E-05 |
| rs79541578 | 17 | 46102728 | 8.3E-26 | 3.2E-07 |
| rs72823599 | 17 | 46132424 | 4.6E-24 | 1.0E-07 |
| rs72823601 | 17 | 46135208 | 6.4E-24 | 1.2E-07 |
| rs72825504 | 17 | 46139479 | 6.8E-24 | 1.3E-07 |
| rs76950846 | 17 | 46102153 | 7.2E-24 | 5.3E-08 |
| rs112296021 | 17 | 46101460 | 9.7E-24 | 1.4E-08 |
| rs72823527 | 17 | 46098810 | 1.7E-23 | 9.4E-09 |
| rs113403571 | 17 | 46097524 | 1.8E-23 | 1.3E-08 |
| rs41280120 | 17 | 46114692 | 1.9E-23 | 1.9E-08 |
| rs72823530 | 17 | 46109371 | 1.9E-23 | 2.0E-08 |
| rs8073371 | 17 | 46096276 | 2.2E-23 | 1.5E-08 |
| rs8067005 | 17 | 46119775 | 3.4E-23 | 1.9E-08 |
| rs72825517 | 17 | 46171824 | 4.2E-23 | 2.7E-07 |
| rs72823591 | 17 | 46122628 | 4.8E-23 | 4.9E-08 |
| rs72825512 | 17 | 46155069 | 9.4E-23 | 1.6E-07 |
| rs7211143 | 17 | 46547381 | 1.4E-22 | 2.4E-06 |
| rs16953235 | 17 | 46244948 | 1.5E-22 | 2.1E-07 |
| rs41336845 | 17 | 46215110 | 1.7E-22 | 3.4E-07 |
| rs16954204 | 17 | 46315198 | 1.7E-22 | 6.9E-07 |
| rs72825548 | 17 | 46231108 | 2.0E-22 | 3.3E-07 |
| rs16954078 | 17 | 46306127 | 2.2E-22 | 3.4E-07 |
| rs112021404 | 17 | 46306843 | 2.5E-22 | 3.4E-07 |
| rs72823594 | 17 | 46124276 | 2.6E-22 | 1.0E-06 |
| rs16953461 | 17 | 46261736 | 2.6E-22 | 3.3E-07 |
| rs16953925 | 17 | 46294498 | 3.0E-22 | 4.0E-07 |
| rs16953382 | 17 | 46252906 | 3.3E-22 | 3.1E-07 |
| rs116944299 | 17 | 46267765 | 3.4E-22 | 4.5E-07 |
| rs72825584 | 17 | 46293089 | 3.8E-22 | 4.0E-07 |
| rs74992250 | 17 | 46272986 | 4.1E-22 | 3.8E-07 |
| rs10514938 | 17 | 46322604 | 4.9E-22 | 3.5E-07 |
| rs112306902 | 17 | 46191032 | 1.2E-21 | 1.7E-07 |
| rs1050025 | 17 | 46148155 | 1.9E-21 | 1.4E-07 |
| rs16952265 | 17 | 46184883 | 2.1E-21 | 3.0E-07 |
| rs58848175 | 17 | 46043282 | 5.8E-19 | 8.8E-05 |
| rs16955073 | 17 | 46381746 | 9.1E-19 | 1.4E-08 |
| rs111750009 | 17 | 46342596 | 1.4E-18 | 6.1E-08 |
| rs72827844 | 17 | 46434441 | 1.5E-18 | 1.7E-08 |
| rs16955463 | 17 | 46399497 | 1.5E-18 | 1.4E-08 |
| rs16954581 | 17 | 46359502 | 1.6E-18 | 3.8E-08 |
| rs72827858 | 17 | 46474361 | 1.8E-18 | 1.2E-08 |
| rs57583172 | 17 | 46481963 | 2.4E-18 | 2.0E-08 |
| rs140817062 | 17 | 46347877 | 2.4E-18 | 9.1E-08 |
| rs72827808 | 17 | 46352080 | 6.2E-18 | 1.3E-08 |
| rs17695373 | 17 | 46375467 | 5.4E-17 | 4.6E-07 |
| rs113791413 | 17 | 46351592 | 5.4E-17 | 1.2E-06 |
| rs72827830 | 17 | 46397502 | 6.8E-17 | 4.2E-07 |
| rs72827814 | 17 | 46366960 | 9.4E-17 | 4.1E-07 |
| rs72827877 | 17 | 46524904 | 9.9E-17 | 4.8E-07 |
| rs72827876 | 17 | 46523974 | 1.0E-16 | 6.1E-07 |
| rs17624626 | 17 | 46535805 | 1.1E-16 | 7.0E-07 |
| rs1472966 | 17 | 46430145 | 1.1E-16 | 7.6E-07 |
| rs72827832 | 17 | 46407108 | 1.1E-16 | 4.5E-07 |
| rs72827834 | 17 | 46413819 | 1.2E-16 | 3.4E-07 |
| rs17696392 | 17 | 46427160 | 1.4E-16 | 3.8E-07 |
| rs72827882 | 17 | 46532624 | 1.6E-16 | 3.6E-07 |
| rs72827885 | 17 | 46534423 | 1.9E-16 | 7.3E-07 |
| rs72827887 | 17 | 46537636 | 1.9E-16 | 5.6E-07 |
| rs78250051 | 17 | 46545826 | 2.9E-16 | 5.3E-07 |
| rs72827864 | 17 | 46492878 | 4.7E-16 | 4.9E-08 |
| rs72827839 | 17 | 46420996 | 5.8E-16 | 1.0E-06 |
| rs72827901 | 17 | 46554456 | 6.3E-16 | 5.8E-07 |
| rs72827899 | 17 | 46551866 | 6.4E-16 | 4.4E-07 |
| rs79799256 | 17 | 46554341 | 7.3E-16 | 4.8E-07 |
| rs59386258 | 17 | 46499918 | 7.9E-16 | 2.8E-08 |
| rs16956892 | 17 | 46523919 | 9.8E-16 | 2.9E-08 |
| rs58953228 | 17 | 46513814 | 1.2E-15 | 6.0E-08 |
| rs4794333 | 17 | 46045495 | 7.8E-13 | 6.8E-09 |
| rs886444 | 17 | 46051911 | 1.4E-12 | 9.1E-09 |
| rs885635 | 17 | 46072228 | 2.6E-09 | 2.2E-08 |
| rs117972490 | 17 | 46046649 | 3.2E-09 | 1.9E-08 |
| rs11650615 | 17 | 46123698 | 6.3E-09 | 2.2E-08 |
| rs61454280 | 17 | 46050125 | 6.3E-09 | 5.5E-08 |
| rs8067216 | 17 | 46088197 | 8.5E-08 | 1.6E-08 |

Supplementary table 2: The GGE GWAS signal encompassing 84 eQTL PNPO SNPs were tested for association with the five vitamin B6 metabolite measures. Nominally significant associations (uncorrected P<0.05) are displayed; these associations are not significant after correction for multiple tests.

| **Vitamin B6 phenotype** | **SNP** | **Chromosome** | **Position** | **Vitamin B6 GWAS P-value** |
| --- | --- | --- | --- | --- |
| PAr index | rs16952265 | 17 | 46184883 | 0.048351 |
| PAr index | rs112306902 | 17 | 46191032 | 0.043809 |
| PAr index | rs41336845 | 17 | 46215110 | 0.040055 |
| PAr index | rs72825548 | 17 | 46231108 | 0.0411 |
| PAr index | rs16953235 | 17 | 46244948 | 0.048542 |
| PAr index | rs16953382 | 17 | 46252906 | 0.042702 |
| PAr index | rs74992250 | 17 | 46272986 | 0.048711 |
| PAr index | rs72825584 | 17 | 46293089 | 0.048388 |
| PAr index | rs16953925 | 17 | 46294498 | 0.048388 |
| PAr index | rs16954078 | 17 | 46306127 | 0.048388 |
| PAr index | rs112021404 | 17 | 46306843 | 0.048388 |
| PAr index | rs16954204 | 17 | 46315198 | 0.048388 |
| PAr index | rs10514938 | 17 | 46322604 | 0.044798 |
| PAr index | rs72827882 | 17 | 46532624 | 0.048397 |
| PAr index | rs72827885 | 17 | 46534423 | 0.048397 |
| PAr index | rs17624626 | 17 | 46535805 | 0.047935 |
| PAr index | rs72827887 | 17 | 46537636 | 0.049912 |
| PAr index | rs7211143 | 17 | 46547381 | 0.02214 |
| PLP:PL | rs79541578 | 17 | 46102728 | 0.04582 |
| PLP:PL | rs72823591 | 17 | 46122628 | 0.044365 |

Supplementary table 3: Genome-wide significant signals in ALPL and PYROXD2 were tested for association with GGE. These associations are not significant after correction for multiple tests.

| **Locus** | **SNP** | **Chromosome** | **Position** | **GGE GWAS P-value** |
| --- | --- | --- | --- | --- |
| ALPL | rs1827293 | 1 | 21795388 | 0.6617 |
| ALPL | rs10737459 | 1 | 21780834 | 0.12 |
| ALPL | rs2016203 | 1 | 21779737 | 0.11 |
| ALPL | rs4021228 | 1 | 21863691 | 0.3543 |
| ALPL | rs1780324 | 1 | 21821757 | 0.7788 |
| ALPL | rs1256327 | 1 | 21897776 | 0.1111 |
| ALPL | rs1256343 | 1 | 21874843 | 0.1977 |
| ALPL | rs10799692 | 1 | 21775943 | 0.13 |
| ALPL | rs10737457 | 1 | 21776392 | 0.16 |
| ALPL | rs1780322 | 1 | 21823076 | 0.6663 |
| ALPL | rs1697422 | 1 | 21821897 | 0.7117 |
| ALPL | rs10799701 | 1 | 21820990 | 0.69 |
| ALPL | rs10799699 | 1 | 21820961 | 0.672 |
| ALPL | rs1780320 | 1 | 21899491 | 0.07414 |
| ALPL | rs35488260 | 1 | 21900459 | 0.07101 |
| ALPL | rs1256329 | 1 | 21895977 | 0.05023 |
| ALPL | rs4654748 | 1 | 21786068 | 0.6001 |
| ALPL | rs3820296 | 1 | 21806447 | 0.8 |
| ALPL | rs1697421 | 1 | 21823292 | 0.3737 |
| ALPL | rs2004380 | 1 | 21797996 | 0.5947 |
| ALPL | rs35331929 | 1 | 21854526 | 0.08307 |
| ALPL | rs1256349 | 1 | 21856616 | 0.1054 |
| ALPL | rs10737456 | 1 | 21776121 | 0.17 |
| ALPL | rs6687836 | 1 | 21796770 | 0.6425 |
| ALPL | rs1256350 | 1 | 21856358 | 0.07656 |
| ALPL | rs1256331 | 1 | 21895008 | 0.04378 |
| ALPL | rs1106357 | 1 | 21817085 | 0.5797 |
| ALPL | rs1256334 | 1 | 21891734 | 0.1382 |
| ALPL | rs2016464 | 1 | 21778965 | 0.16 |
| ALPL | rs1256338 | 1 | 21889167 | 0.1691 |
| ALPL | rs6426713 | 1 | 21803177 | 0.6056 |
| ALPL | rs6685071 | 1 | 21776884 | 0.14 |
| ALPL | rs10799700 | 1 | 21820968 | 0.7203 |
| ALPL | rs2016413 | 1 | 21779465 | 0.1 |
| ALPL | rs1772719 | 1 | 21904374 | 0.9771 |
| ALPL | rs1780329 | 1 | 21902950 | 0.016 |
| ALPL | rs12726396 | 1 | 21878934 | 0.1656 |
| ALPL | rs6656758 | 1 | 21897961 | 0.7134 |
| ALPL | rs1256346 | 1 | 21861108 | 0.1373 |
| ALPL | rs1256335 | 1 | 21890386 | 0.3432 |
| ALPL | rs1780316 | 1 | 21889635 | 0.3562 |
| ALPL | rs2275370 | 1 | 21900420 | 0.7785 |
| ALPL | rs2010202 | 1 | 21780651 | 0.12 |
| ALPL | rs10799702 | 1 | 21821880 | 0.9811 |
| PYROXD2 | rs942813 | 10 | 100153608 | 0.1677 |
| PYROXD2 | rs11189590 | 10 | 100154605 | 0.3232 |
| PYROXD2 | rs7096654 | 10 | 100134036 | 0.7312 |
| PYROXD2 | rs4539242 | 10 | 100148058 | 0.2214 |
| PYROXD2 | rs2182168 | 10 | 100148353 | 0.13 |
| PYROXD2 | rs2182169 | 10 | 100148004 | 0.1653 |
| PYROXD2 | rs10786418 | 10 | 100157368 | 0.2122 |
| PYROXD2 | rs4551689 | 10 | 100157593 | 0.09 |
| PYROXD2 | rs7075655 | 10 | 100142068 | 0.07267 |
| PYROXD2 | rs7082192 | 10 | 100151844 | 0.11 |
| PYROXD2 | rs1079179 | 10 | 100149346 | 0.11 |
| PYROXD2 | rs2147896 | 10 | 100148176 | 0.2214 |
| PYROXD2 | rs11819729 | 10 | 100157726 | 0.1589 |
| PYROXD2 | rs4488133 | 10 | 100159136 | 0.1074 |
| PYROXD2 | rs11189587 | 10 | 100154545 | 0.5469 |
| PYROXD2 | rs7916469 | 10 | 100148653 | 0.11 |
| PYROXD2 | rs11597425 | 10 | 100154202 | 0.1939 |
| PYROXD2 | rs7907555 | 10 | 100155810 | 0.2303 |
| PYROXD2 | rs11189586 | 10 | 100154096 | 0.2289 |
| PYROXD2 | rs7072216 | 10 | 100156853 | 0.1214 |
| PYROXD2 | rs7924303 | 10 | 100155963 | 0.2141 |
| PYROXD2 | rs11189589 | 10 | 100154565 | 0.5232 |
| PYROXD2 | rs1739 | 10 | 100176339 | 0.3103 |
| PYROXD2 | rs942814 | 10 | 100151305 | 0.1849 |
| PYROXD2 | rs1737 | 10 | 100176366 | 0.24 |
| PYROXD2 | rs7909131 | 10 | 100171471 | 0.3379 |
| PYROXD2 | rs2274247 | 10 | 100150148 | 0.094 |
| PYROXD2 | rs4400721 | 10 | 100147097 | 0.07416 |
| PYROXD2 | rs3830020 | 10 | 100176615 | 0.3948 |
| PYROXD2 | rs6584191 | 10 | 100152055 | 0.13 |
| PYROXD2 | rs11597428 | 10 | 100154247 | 0.1927 |
| PYROXD2 | rs7097824 | 10 | 100151799 | 0.11 |
| PYROXD2 | rs7073632 | 10 | 100160846 | 0.12 |
| PYROXD2 | rs755126 | 10 | 100148951 | 0.11 |
| PYROXD2 | rs7896828 | 10 | 100170507 | 0.3497 |
| PYROXD2 | rs7924209 | 10 | 100155927 | 0.1887 |
| PYROXD2 | rs10786414 | 10 | 100141027 | 0.1738 |
| PYROXD2 | rs10883087 | 10 | 100156990 | 0.1989 |
| PYROXD2 | rs7905265 | 10 | 100143193 | 0.1775 |
| PYROXD2 | rs11189581 | 10 | 100137700 | 0.7474 |
| PYROXD2 | rs1061123 | 10 | 100176997 | 0.359 |
| PYROXD2 | rs6584192 | 10 | 100152373 | 0.11 |
| PYROXD2 | rs10883084 | 10 | 100151189 | 0.11 |
| PYROXD2 | rs10786415 | 10 | 100151266 | 0.1907 |
| PYROXD2 | rs942810 | 10 | 100167860 | 0.3299 |
| PYROXD2 | rs1061115 | 10 | 100177049 | 0.3171 |
| PYROXD2 | rs2147895 | 10 | 100148308 | 0.13 |
| PYROXD2 | rs4345897 | 10 | 100147060 | 0.1232 |
| PYROXD2 | rs10883086 | 10 | 100155743 | 0.257 |
| PYROXD2 | rs7097625 | 10 | 100158922 | 0.1352 |
| PYROXD2 | rs11189591 | 10 | 100155296 | 0.1553 |
| PYROXD2 | rs2296438 | 10 | 100146895 | 0.1665 |
| PYROXD2 | rs2147897 | 10 | 100147813 | 0.1527 |
| PYROXD2 | rs10883085 | 10 | 100155613 | 0.17 |
| PYROXD2 | rs7897357 | 10 | 100163597 | 0.3856 |
| PYROXD2 | rs6584194 | 10 | 100160399 | 0.134 |
| PYROXD2 | rs10786416 | 10 | 100153600 | 0.2246 |

Supplementary table 4: Logistic regression to assess the difference in pyridoxine-related metabolite PRS scores between people with GGE compared to controls. None of the associations reached the significance threshold of P<0.001 that is recommended for analyses with PRSice.

| **Metabolite** | **P-threshold** | **Number of SNPs** | **Explained variance** | **Standardized beta** | **Standard Error** | **P-value** |
| --- | --- | --- | --- | --- | --- | --- |
| PLP | 0.0034 | 823 | 0.00035 | -0.042 | 0.020 | 0.03 |
| PL | 0.0003 | 103 | 0.00067 | -0.058 | 0.020 | 0.003 |
| PA | 0.0063 | 1532 | 0.00020 | 0.033 | 0.020 | 0.10 |
| PLP:PL | 0.0001 | 35 | 0.00070 | 0.060 | 0.020 | 0.002 |
| PAr index | 0.0090 | 2068 | 0.00067 | 0.058 | 0.019 | 0.003 |
